# Cardiovascular Consequences of E-Cigarettes: A New Challenge for Cardiologists

**DOI:** 10.3390/jcm15062226

**Published:** 2026-03-15

**Authors:** Florin-Dumitru Mihălțan, Ruxandra Ulmeanu, Armand Râjnoveanu, Ancuța-Alina Constantin

**Affiliations:** 1 Department of Cardio-Thoracic Pathology, “Carol Davila” University of Medicine and Pharmacy, 050474 Bucharest, Romania; florin.mihaltan@umfcd.ro; 2 Institute of Pneumology “Marius Nasta”, 050159 Bucharest, Romania; 3 Doctoral School of Biomedical Sciences, Faculty of Medicine and Pharmacy, University of Oradea, 410073 Oradea, Romania; 4 Occupational Medicine Department, Iuliu Hatieganu University of Medicine and Pharmacy, 400012 Cluj-Napoca, Romania

**Keywords:** electronic cigarette, cardiovascular diseases, e-cigarette

## Abstract

The electronic cigarette has divided the medical community. While promoted as a tool for smoking cessation, its use has opened a Pandora’s box. Evidence from studies on both cardiovascular and pulmonary health demonstrates that e-cigarettes are associated with multiple adverse effects. In this review, we specifically examine their consequences and associations with coronary artery disease, myocardial infarction, arrhythmias, arterial hypertension, and related conditions. Finally, we highlight approaches to counter the spread of these so-called harm-reduction alternatives, drawing on data from the European Respiratory Society, the European Society of Cardiology, and the Cochrane Collaboration.

## 1. Introduction

Electronic cigarettes (e-cigarettes) are battery-powered devices that aerosolize e-liquids, typically composed of propylene glycol (PG), vegetable glycerin (VG), nicotine, flavorings, and stabilizers or humectants such as triacetin [1]. More than 8000 unique flavors and over 450 brands of e-cigarettes have been reported, with limited but growing evidence suggesting that many of these substances may be detrimental to human health [2].

Initially regarded as a fashionable gadget, e-cigarettes quickly gained popularity both as a potential aid for tobacco smoking cessation and as a substitute for traditional cigarettes [3]. Observational cohort confirmed and reported higher smoking rates among e-cigarette users at 12 months, while concerns persist regarding continued e-cigarette use and youth progression to combustible tobacco [4]. Although they do not involve combustion and therefore produce no carbon monoxide, trace levels of nitrosamines, formaldehyde, acetaldehyde, and heavy metals have been detected—albeit in lower quantities than in conventional cigarettes. E-cigarette use among 13–15-year-olds in the WHO European Region is now among the highest globally, with recent reports describing an alarming increase in youth vaping despite progress in tobacco control [5]. Since their commercial introduction 17 years ago, debate has persisted on two major fronts: the health risks associated with e-cigarette use and their potential utility as tools for smoking cessation. From the outset, a race has unfolded between the tobacco industry, which promotes their benefits, and researchers and clinicians, who remain divided over their harms versus their therapeutic potential.

In this narrative review, we aim to synthesize current evidence on the cardiovascular consequences of electronic cigarette use, with a focus on coronary artery disease, myocardial infarction, arrhythmias, arterial hypertension, and related conditions. Our objective is to critically evaluate both experimental and clinical data to clarify whether e-cigarettes represent a safer alternative to conventional smoking from a cardiovascular standpoint and to provide evidence-based guidance for clinicians and policymakers.

Clinicians—particularly cardiologists—often struggle with whether to recommend e-cigarettes as a means of reducing traditional cigarette consumption. A common clinical question is: “Doctor, should I switch to electronic cigarettes? Are they less harmful?”

The first challenge lies in terminology. The inaccurate use of terms such as “e-cigarette smoking” in academic literature has important implications for addiction science, public health, and policy. Conflating vaping with smoking obscures the unique mechanisms of nicotine dependence, complicates harm-reduction strategies, and risks misinforming both clinicians and the public [6].

## 2. Methods

This article was conducted as a narrative review. Unlike a systematic review, it does not aim to exhaustively identify all available evidence through a predefined protocol but rather to provide a structured and critical synthesis of the most relevant literature. Given the rapidly evolving landscape of e-cigarette technologies and cardiovascular research, a narrative approach was considered appropriate to integrate mechanistic, experimental, and clinical data and to contextualize findings for practicing clinicians and policymakers. A targeted search of electronic databases, including PubMed/MEDLINE and Scopus, was performed to identify relevant studies published primarily between 2010 and September 2025. Additional references were identified through manual screening of bibliographies of selected articles and position statements from major professional societies, including the European Respiratory Society, the European Society of Cardiology, the European Association of Preventive Cardiology, and the Cochrane Collaboration.

We prioritized peer-reviewed original research articles, systematic reviews, meta-analyses, randomized controlled trials, large observational cohort studies, and mechanistic experimental studies addressing cardiovascular outcomes associated with e-cigarette use. Key search terms included combinations of: “electronic cigarettes,” “e-cigarettes,” “vaping,” “cardiovascular disease,” “myocardial infarction,” “arrhythmia,” “heart failure,” “hypertension,” “stroke,” “endothelial dysfunction,” and “oxidative stress.”

Studies focusing exclusively on pulmonary outcomes without cardiovascular relevance were excluded unless directly related to cardiopulmonary interactions. Non-peer-reviewed sources were not included, with the exception of major institutional or regulatory reports deemed relevant to clinical practice or public health policy.

Given the narrative design, no formal PRISMA framework, quantitative synthesis, or predefined risk-of-bias assessment tool was applied. While this approach allows for broader conceptual integration of diverse evidence types, it may introduce selection bias. We therefore aimed to mitigate this limitation by prioritizing high-quality studies and transparently acknowledging areas of uncertainty and conflicting findings.

## 3. Components of E-Cigarettes and Acute and Chronic Cardiovascular Effects

The E-cigarette aerosols contain far fewer chemicals than conventional cigarettes. They typically include propylene glycol, vegetable glycerin, lower levels of oxidants and volatile organic compounds, minimal or no carbon monoxide, liquid particles of unknown toxicity, various metals, nicotine, and flavoring agents. Notably, the quantity of microparticles detected in e-cigarette aerosols is comparable to that found in conventional cigarettes [7]. At higher operating temperatures, thermal degradation of propylene glycol and vegetable glycerin generates toxic volatile organic compounds such as acrolein, formaldehyde, and acetaldehyde. Flavoring chemicals—including diacetyl, cinnamaldehyde, vanillin acetals, and other compounds—also raise significant toxicity concerns [8,9].

The nicotine delivered by e-cigarettes varies substantially depending on several factors: nicotine concentration in the e-liquid, user experience, puffing intensity, and device characteristics. First-generation devices typically deliver less nicotine compared with more recent designs [10]. Nicotine itself plays a paradoxical role—serving as the principal component in harm-reduction products while simultaneously exerting harmful effects on the cardiovascular system (CVS). Specifically, nicotine impairs vascular function and promotes vascular calcification [11].

Flavoring agents present additional risks. Flavored e-cigarettes are not harmless; experimental studies suggest they may contribute to cardiac injury. For example, vanillin aldehyde-flavored vapor exposure significantly increased ventricular action potential duration alternans in mice compared with controls [12]. Likewise, menthol-flavored aerosol exposure induced basal sinus bradycardia persisting for up to three weeks post-exposure, along with an altered chronotropic response to restraint stress and prolonged ventricular repolarization (QTc interval) [13].

Human and animal studies also provide evidence of acute hemodynamic changes. E-cigarette use acutely increases heart rate (HR), systolic blood pressure (SBP), and diastolic blood pressure (DBP) [11]. Arterial stiffness, measured by carotid–femoral pulse wave velocity, rises within five minutes of use. In contrast, chronic substitution of tobacco with e-cigarettes did not significantly affect HR but was associated with reductions in both SBP and DBP [11].

**Figure 1:**
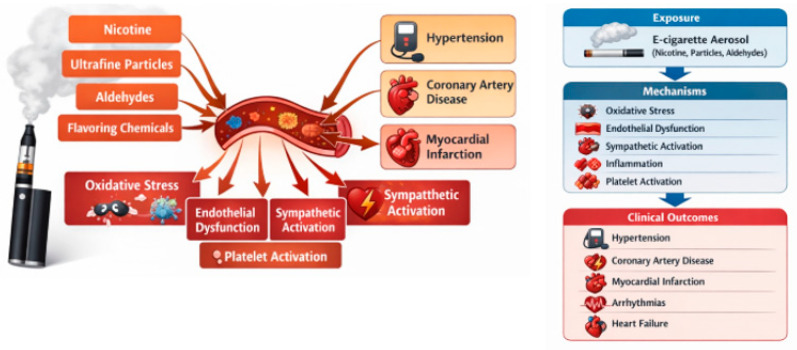
Proposed mechanisms linking e-cigarette use to CVD.

E-cigarettes also impair endothelial function, similar to conventional cigarettes. They reduce nitric oxide bioavailability [14] and increase circulating endothelial progenitor cells, likely reflecting acute endothelial dysfunction and vascular injury (Figure 1). Other reported effects include elevated oxidative stress, a shift in cardiac autonomic balance toward sympathetic predominance [15], increased cardiac sympathetic nerve activity [16], and heightened risks of thrombosis and atherosclerosis [17].

Additional consequences include hyperlipidemia, sympathetic dominance, endothelial dysfunction, DNA damage, and macrophage activation. Oxidative stress and inflammation appear to be unifying mechanisms underlying cardiovascular impairment associated with e-cigarette exposure [18]. Chronic exposure in animal models has been linked to increased arterial stiffness, endothelial alterations, angiogenesis, cardiorenal fibrosis, and accelerated atherosclerotic plaque formation [19,20].

Beyond acute endothelial and autonomic effects, several pathways implicated in adverse remodeling and heart failure—particularly TGF-β-driven cardiac fibroblast activation—may represent plausible downstream mechanisms linking chronic exposure to aerosol constituents with myocardial fibrosis. Experimental evidence demonstrates that xanthohumol inhibits TGF-β1-induced cardiac fibroblast activation via modulation of the PTEN/Akt/mTOR signaling pathway, thereby attenuating collagen synthesis and fibrotic remodeling [21]. Although this anti-fibrotic mechanism was not investigated in the context of e-cigarette exposure, it provides a relevant comparative framework, suggesting that vaping-related oxidative stress and inflammatory signaling could theoretically converge on similar pro-fibrotic cascades. Direct confirmation of TGF-β pathway activation in chronic e-cigarette users remains necessary.

Overall, the most robust data concern short-term (months to years) outcomes, particularly from human studies of acute (minutes to hours) and sub-acute (weeks to months) e-cigarette exposure.

## 4. E-Cigarettes and Cardiovascular Diseases

The relationship between e-cigarette use and cardiovascular disease (CVD) remains complex, with conflicting evidence. Two large cross-sectional studies reported no significant association between exclusive e-cigarette use and CVD [22,23]. However, most research highlights adverse cardiovascular effects. Berlowitz et al. [24], in a longitudinal analysis of the PATH Study, reported that cardiovascular disease (CVD) risk among exclusive e-cigarette users appeared lower than that observed in conventional cigarette smokers; however, this reduction reached statistical significance only for the composite outcome of “any CVD.” Importantly, the study did not demonstrate a statistically significant increase in CVD risk among exclusive e-cigarette users compared with non-users. These findings must be interpreted with caution, as the number of cardiovascular events among exclusive e-cigarette users was relatively small (n = 20), limiting statistical power and precision of the estimates. Consequently, the absence of a significant association should not be interpreted as evidence of cardiovascular safety, but rather as reflecting insufficient event numbers to draw firm conclusions. In contrast, dual users exhibited risks comparable to exclusive combustible cigarette smokers for overall CVD, myocardial infarction, heart failure, and stroke. Larger studies with longer follow-up and greater numbers of outcome events are needed to more definitively clarify the cardiovascular risk profile of exclusive e-cigarette use.

Importantly, the evidence base is continuing to evolve, and more recent large datasets have begun to provide signals relevant to longer-term outcomes. In 2024, analyses from the NIH “All of Us” Research Program reported that ever-use of e-cigarettes was associated with a higher risk of incident heart failure over a median follow-up of approximately four years, with the association appearing strongest for heart failure with preserved ejection fraction (HFpEF). Although observational in nature and not proof of causality, these findings add prospective evidence supporting concern about potential chronic cardiovascular consequences of vaping [25].

In parallel, a 2024 meta-analysis synthesizing observational studies through April 2024 reported that dual use and e-cigarette use in individuals with prior combustible cigarette exposure were associated with increased odds of CVD, whereas exclusive current e-cigarette use was not statistically significantly associated with CVD (with confidence intervals consistent with potential harm but limited precision). This pattern reinforces a key interpretive point: the absence of statistical significance in exclusive-user analyses should not be interpreted as evidence of cardiovascular safety, but rather as reflecting ongoing uncertainty and the need for larger longitudinal studies with sufficient outcome events and careful control for smoking history and dual use [26].

Other studies present even more concerning findings. For example, one analysis suggested that individuals who use e-cigarettes in an attempt to taper combustible cigarette consumption do not reduce their CVD risk compared to exclusive smokers [27]. Similarly, cross-sectional evaluations of cardiovascular symptoms have demonstrated that e-cigarette users face a higher risk of coronary heart disease, arrhythmia, chest pain, and palpitations [28]. Taken together, current evidence indicates that exclusive e-cigarette use may not be harmless and that dual use confers no cardiovascular benefit. Recent prospective and pooled observational evidence (including 2024 analyses) continues to support caution regarding longer-term outcomes, while also highlighting that risk estimates for exclusive users remain imprecise and sensitive to prior smoking history and dual use. Moreover, while e-cigarettes are often promoted as a harm-reduction tool, their use may still contribute significantly to cardiopulmonary health risks, particularly in relation to respiratory outcomes.

The clinical manifestations of cardiovascular injury associated with e-cigarette exposure span multiple domains, including atherosclerotic disease, myocardial injury, electrical instability, heart failure, and vascular dysfunction.

### 4.1. Coronary Artery Disease

The constituents of e-cigarettes—including many of the same components found in conventional cigarettes—promote systemic inflammation and oxidative stress, directly affecting blood vessels such as the coronary arteries [24]. Nicotine also plays a complex role in coronary physiology: it decreases coronary blood flow by stimulating vascular smooth muscle α1-adrenergic receptors, causing vasoconstriction. At the same time, findings from human studies indicate that nicotine can increase coronary blood flow by enhancing cardiac output, inducing subsequent flow-mediated dilation (FMD), and directly stimulating β_2_-receptors in coronary arteries, leading to vasodilation [29]. Although e-liquids contain fewer chemicals than combustible cigarettes, some constituents still pose substantial risks to cardiovascular health. For example, Busch et al. [30] reported that exclusive e-cigarette users perceived their one-year risk of myocardial infarction as 34.6%, which was significantly lower than the perceived risk among tobacco cigarette smokers (56.2%) but markedly higher than that of individuals not using nicotine (15.2%).

### 4.2. Myocardial Infarction

The relationship between e-cigarette use and myocardial infarction (MI) has been clarified by several large-scale studies. A review of nine studies involving 984,764 patients found that e-cigarette users had significantly higher odds of MI compared with non-users [OR 1.44; 95% CI, 1.22–1.74; *p* < 0.0001] [31]. Dual users were at even greater risk, with a more than fourfold increase compared to non-users [OR 4.04; 95% CI, 3.40–4.81; *p* < 0.00001]. These findings confirm that dual use is associated with a higher risk of MI than exclusive e-cigarette use, and that both dual and exclusive use confer greater risk than abstinence [31]. Importantly, the risk increases with greater frequency of e-cigarette consumption. The meta-analysis cited represents a key synthesis of available data on myocardial infarction (MI) risk associated with e-cigarette use. However, it is important to recognize substantial heterogeneity across included studies, stemming from differences in study design (e.g., cross-sectional surveys vs. prospective cohort investigations), definitions of exposure (ever use vs. current use, exclusive vs. dual use), and outcome ascertainment (self-reported vs. clinically adjudicated MI). Such variability can influence pooled effect estimates and complicate causal interpretation. Where subgroup analyses were available, stratification by study design suggested that prospective cohorts tended to show more consistent directionality of associations than cross-sectional studies, though confidence intervals often overlapped. Additional subgroup analyses stratified by exclusive e-cigarette use, duration of use, and adjustment for combustible cigarette history would be valuable to clarify differential risk patterns. Future meta-analyses with prespecified stratification and meta-regression may help disentangle these design-related sources of heterogeneity and provide more nuanced risk estimates.

Overall, e-cigarette use is associated with a 33% higher risk of MI compared to non-use. A separate meta-analysis reported that e-cigarette users have about half the risk of MI compared to conventional cigarette smokers, yet still higher odds than non-users [32]. Mechanistically, exposure to e-cigarette vapor extracts has been shown to enhance platelet activation, adhesion, and aggregation—pathways directly linked to MI [33,34].

Regular e-cigarette use, particularly in combination with conventional cigarette smoking, thus represents a substantial risk factor for MI. Public health messaging should emphasize that e-cigarettes increase the risk of MI, especially with daily use [31]. Evidence also supports a cumulative effect: occasional e-cigarette users had an OR of 1.49 (95% CI, 1.06–2.09), while daily users had an OR of 2.14 (95% CI, 1.41–3.25) [35,36].

Although e-cigarettes have been proposed as tools for smoking cessation, their cardiovascular safety remains uncertain. Some authors have explored their use in cessation strategies following acute coronary syndrome [37]. A network meta-analysis of pharmacological and e-cigarette interventions demonstrated that nicotine-containing e-cigarettes may improve abstinence rates, but raised concerns about cardiovascular safety [38]. Furthermore, myocardial function after infarction was found to be significantly impaired following exposure to nicotine vape aerosol, regardless of sex [39].

### 4.3. Heart Rhythm Disorders

Clinical studies in e-cigarette users have demonstrated sympathoexcitatory effects, raising concerns about their potential role in heart rhythm disorders. Whether these effects are less pronounced than those of conventional tobacco remains uncertain, particularly as e-cigarette technology continues to evolve and alter nicotine pharmacokinetics [40]. Inhalation of nicotine-containing e-cigarette aerosols significantly increases heart rate, arterial stiffness, and airway flow resistance [41]. Acute exposure has been associated with elevated heart rate (HR) (mean difference [MD] 11.329, *p* < 0.01) and blood pressure (BP) (MD 12.856, *p* < 0.01 for systolic; MD 7.676, *p* < 0.01 for diastolic) compared with non-use [42].

Comparative analyses show mixed results: non-smoker current vapers displayed no significant differences in resting HR and BP compared with non-users, but had lower resting HR (MD −2.608, *p* < 0.01) and diastolic BP (MD −3.226, *p* < 0.01) than non-vaper smokers [40]. Nonetheless, nicotine and other constituents in e-cigarettes can provoke arrhythmias or exacerbate pre-existing rhythm disorders. In animal models, acute aerosol inhalation disturbed cardiac conduction partly via parasympathetic modulation [43]. Nicotine may induce both atrial and ventricular arrhythmogenesis, mediated by catecholamine release and potassium channel interactions. Structural remodeling also contributes: nicotine downregulates microRNAs 133 and 590—post-transcriptional repressors of growth factors—thereby promoting atrial fibrillation [44]. While preclinical studies provide important mechanistic insight into the pro-arrhythmic potential of nicotine and other e-cigarette constituents—including parasympathetic modulation, catecholamine release, ion channel interactions, and microRNA dysregulation—direct clinical translation remains limited. The majority of mechanistic data supporting atrial and ventricular arrhythmogenesis derives from controlled animal or in vitro models, in which exposure conditions and nicotine delivery kinetics may differ substantially from real-world human vaping patterns. In contrast, human data are largely observational and focus primarily on surrogate markers such as heart rate variability, QT interval changes, sympathetic predominance, or self-reported palpitations, rather than adjudicated arrhythmic events. Robust prospective studies evaluating incident atrial fibrillation, ventricular arrhythmias, or sudden cardiac death in exclusive e-cigarette users are currently lacking. Therefore, although biological plausibility is strong, the magnitude of clinical arrhythmic risk attributable to e-cigarette use in humans remains incompletely defined and warrants further longitudinal investigation.

Beyond nicotine, solvents such as vegetable glycerin and propylene glycol have been shown to induce bradycardia, bradyarrhythmias, and changes in heart rate variability during exposure, with inverse effects post-exposure. Menthol- and propylene glycol-based aerosols further increase ventricular arrhythmias and augment early ventricular repolarization (J-wave amplitude). Menthol uniquely alters atrial and atrioventricular conduction. These constituents—along with acrolein, formaldehyde, and flavoring chemicals—likely contribute to pro-arrhythmic changes and autonomic imbalance [45].

Clinical studies reinforce these risks. Cross-sectional analyses indicate that e-cigarette users are at higher risk of coronary heart disease, arrhythmia, chest pain, and palpitations compared to non-users [28]. Dual users, in particular, appear more vulnerable to arrhythmias and breathing difficulties, reflecting either the effects of e-cigarettes alone or additive harms from dual exposure [28]. Chronic e-cigarette use has also been associated with QT prolongation [46]. For individuals with long QT syndrome, this may translate into an elevated risk of sudden cardiac death, especially among younger users. Importantly, newer-generation e-cigarettes deliver higher nicotine concentrations, potentially magnifying these arrhythmic risks [47].

### 4.4. Heart Failure

In 2024, MedStar Health researchers analyzed data from the All of Us NIH database and reported a 19% increased risk of HF among ever-users of e-cigarettes. Preliminary data suggest a possible association between e-cigarette use and an increased risk of developing heart failure (HF) with preserved ejection fraction, even after adjusting for traditional risk factors. Several biological mechanisms may underlie this relationship, including nicotine-induced autonomic dysregulation, oxidative stress, inflammation, endothelial dysfunction, and myocardial fibrosis. E-cigarette use has also been linked to increased resting heart rate, an independent risk factor for HF [25,48,49]. Although the associations observed are clinically intriguing and hypothesis-generating, they should be interpreted cautiously. These findings derive from observational data and, while adjusted for multiple traditional cardiovascular risk factors, remain subject to residual confounding, reverse causation, and potential misclassification of exposure or outcome. Moreover, duration and intensity of e-cigarette use were not uniformly characterized, and prior combustible cigarette exposure may not be fully accounted for in all analyses. As such, these results do not establish causality but rather highlight a possible signal that warrants further investigation. Large-scale, prospective cohort studies with detailed exposure assessment, longer follow-up, and adjudicated heart failure outcomes—ideally complemented by mechanistic translational research—are needed to determine whether e-cigarette use independently contributes to incident heart failure.

Emerging data in heart failure research highlight a role for regulated necrotic cell death programs, particularly necroptosis, in disease progression. Transcriptomic analyses have linked necroptosis-related gene signatures with immune cell infiltration patterns and inflammatory amplification in heart failure cohorts [50]. While these studies are not specific to e-cigarette exposure, they suggest a mechanistic bridge whereby repeated exposure to pro-oxidant aerosol constituents may contribute to cardiomyocyte injury, immune activation, and fibrotic remodeling in susceptible individuals. Further translational research is needed to determine whether vaping-related oxidative stress activates similar necroptotic pathways in human myocardium.

Experimental data reinforce these concerns. In obese mice, exposure to e-cigarettes containing 2.4% nicotine produced profound adverse cardiac effects, including increased oxidative stress, elevated plasma free fatty acid levels, cardiomyocyte apoptosis, inactivation of AMP-activated protein kinase, and activation of its downstream target, acetyl-CoA carboxylase [51]. Similarly, another murine study demonstrated that nicotine-containing e-cigarette exposure decreased cardiac fractional shortening and ejection fraction compared with controls. RNA sequencing analysis further revealed a proinflammatory phenotype induced by nicotine-containing aerosols [52]. Together, these findings highlight potential pathways through which e-cigarettes may contribute to the development and progression of HF. However, while these experimental findings provide important mechanistic insights into the potential cardiotoxic effects of specific flavoring agents, caution is warranted when extrapolating results from animal models to humans. Species-specific differences in cardiovascular physiology, autonomic regulation, metabolic processing of inhaled compounds, exposure patterns, and nicotine pharmacokinetics may limit direct translatability. Moreover, experimental exposure conditions in murine models often differ substantially from real-world human vaping behaviors in terms of dose, duration, and device characteristics. Therefore, although animal studies are valuable for identifying biological pathways of injury, their findings should be interpreted as hypothesis-generating and require confirmation in well-designed human studies.

### 4.5. Hypertension

The association between e-cigarette use and hypertension has been increasingly reported in human studies. As a modifiable risk factor, its contribution to elevated blood pressure should not be overlooked. The hypertensive effects observed among e-cigarette users are largely attributable to nicotine, which stimulates the release of norepinephrine and epinephrine, activates the sympathetic nervous system, and increases plasma nicotine concentrations [53]. In a study including 11,593 participants with available e-cigarette use data (mean age 47 years; 52.2% female) followed for six years, ever use of e-cigarettes was associated with an increased risk of incident hypertension, suggesting potential long-term cardiovascular harm [54].

### 4.6. Stroke

E-cigarette use has also been linked to an elevated risk of stroke. In a large cross-sectional analysis of National Health Interview Survey data, Vindhyal et al. [55] reported a 30% higher odds of stroke among e-cigarette users compared with non-users (OR 1.30; 95% CI, 1.20–1.40). However, as this study was cross-sectional in design, causal inference is inherently limited. Stroke history and e-cigarette use were self-reported, introducing the potential for recall bias and misclassification. Additionally, cross-sectional analyses cannot establish temporal sequence, raising the possibility of reverse causation (e.g., individuals with prior stroke switching to e-cigarettes). Although multivariable-adjusted odds ratios were reported to account for demographic characteristics and conventional cardiovascular risk factors, residual confounding—particularly from prior or concurrent combustible cigarette use—remains possible. Evidence regarding the association between e-cigarette use and stroke remains inconclusive. In a recent network meta-analysis, cigarette smoking and dual use of cigarettes and e-cigarettes were significantly associated with increased odds of stroke compared with never use, whereas e-cigarette use alone was not significantly associated with stroke risk (AOR 1.44; 95% CI 0.92–2.24). Dual use ranked highest for stroke risk, followed by cigarette smoking, while exclusive e-cigarette use showed an intermediate but non-significant association. Although no statistically significant relationship between e-cigarette use and stroke was identified, a trend toward increased risk was observed, consistent with findings from previous meta-analyses. Differences between studies may reflect variations in study design, sample size, and whether dual users were analyzed separately from exclusive e-cigarette users [56].

Future prospective cohort studies with adjudicated stroke outcomes and detailed exposure characterization are needed to clarify whether e-cigarette use independently increases stroke risk.

### 4.7. Other General Cardiovascular Effects

Espinoza-Derout et al. demonstrated increased atherosclerosis in the aortic root of mice exposed to nicotine-containing e-cigarettes, further supporting the presence of a proinflammatory phenotype associated with cardiac dysfunction and atherogenesis [52].

Associations have also been reported between vaping and pro-thrombotic biomarkers. Experimental studies indicate that vaping acutely induces platelet aggregation and activation, raising concerns about its potential role in thrombogenesis. The long-term consequences of chronic e-cigarette use on thrombosis risk remain insufficiently studied [32,57]. In addition, oxidant chemicals and heavy metals present in e-cigarette aerosols may promote cardiovascular disease through several mechanisms, including inflammation, endothelial dysfunction, endothelial cytotoxicity, vascular injury, and thrombotic activation [58].

Beyond direct nicotine-mediated sympathetic stimulation, cardiovascular responses to vaping may also be influenced by receptor-dependent regulatory systems. The G protein-coupled estrogen receptor (GPER) has been increasingly recognized as an important modulator of vascular tone, myocardial remodeling, hypertension, atherosclerosis, myocardial infarction, and arrhythmogenesis [59]. Although direct links between e-cigarette exposure and GPER signaling have not been established, hormonal receptor pathways may contribute to inter-individual variability and potential sex-specific differences in cardiovascular susceptibility to nicotine-containing aerosols.

Insights from environmental cardiovascular toxicology may also inform understanding of vaping-related risks. Recent analyses of tire-derived pollutants, particularly 6PPD and its oxidation product 6PPD-quinone, have identified core molecular targets associated with oxidative stress, vascular dysfunction, and myocardial injury in cardiovascular disease models [60]. Although these pollutants are chemically distinct from e-cigarette aerosols, they illustrate how inhaled environmental toxicants can converge on shared pathogenic pathways relevant to atherosclerosis, myocardial infarction, and heart failure. Such mechanistic analogies underscore the importance of comprehensive molecular profiling of e-cigarette aerosols to clarify long-term cardiovascular implications.

## 5. Prevention Methods to Protect Our Patients

Legislation regarding e-cigarettes remains inconsistent worldwide. Within the European Union, Article 20 of the Tobacco Products Directive permits classification of e-cigarettes as medicines if specific conditions are met. However, there is no consensus on how to regulate sales, packaging, taxation, and public use. As of now, legislation is available in 98 countries and varies considerably: only 13 apply taxes to e-cigarettes, 29 ban them completely, and 9 prohibit nicotine-containing liquids only [61]. Higher prices and restrictions on vaping have been associated with lower rates of e-cigarette use [62].

At the supranational level, electronic cigarettes and related products in the European Union are primarily regulated under the Tobacco Products Directive (2014/40/EU), which entered into force in 2014 and became applicable in all Member States by May 2016. Article 20 of the Directive sets out detailed requirements for the manufacture, presentation, sale, safety and quality of e-cigarettes and refill containers, including maximum nicotine concentrations, labelling, and notification obligations for manufacturers and importers prior to market placement. The Directive also empowers Member States to restrict internet sales and advertising and to implement additional health-oriented measures consistent with public health objectives [63].

Although this Directive remains the cornerstone of EU product regulation for vaping devices, Member States retain significant discretion to adopt national legislation in areas not harmonized at the EU level, such as age limits, smoke-free policies, flavour restrictions beyond those set by the TPD, and marketing controls. Recent assessments of implementation reveal that at least 16 EU Member States have independently introduced bans on online sales or additional restrictions on cross-border distance sales for e-cigarettes, heated tobacco products, and nicotine pouches, reflecting mounting concerns about youth uptake and marketing practices [64].

In addition, there is active discussion among EU Member States and the European Commission about updating the broader tobacco taxation framework to more explicitly encompass vaping products and harmonize excise duties, an initiative that gained political momentum in 2025 with proposals from a coalition of finance ministers urging minimum excise rates for vapes, nicotine pouches and heated tobacco products. Such reforms, if adopted, would further modernize EU tobacco and nicotine policy in response to evolving product markets and public health priorities.

Warning labels also play a role in prevention. Text-only e-cigarette warnings have been shown to increase awareness about the harms and addictiveness of vaping without creating the misperception that e-cigarettes are more harmful than conventional cigarettes. These warnings were also associated with reduced intentions to vape and greater intentions to quit, supporting the inclusion of health risk information in e-cigarette warning labels [65].

Intervention strategies to support vaping cessation remain in their early stages of development. A recent Cochrane review found only low-certainty evidence that text message-based programs and varenicline may help individuals quit vaping [66]. Overall, the available data suggest that existing interventions provide limited long-term success, and sustained cessation remains a significant challenge for most e-cigarette users.

Taken together, these developments illustrate that while the 2014 Directive continues to provide the legislative foundation for e-cigarette regulation in the EU, policy evolution is ongoing, with Member States increasingly advancing complementary measures and the Commission considering updates to address novel products, digital marketing, youth prevention and taxation strategies.

## 6. How Effective Are E-Cigarettes as a Protective Smoking-Cessation Tool for Patients with Cardiac Disease?

There is high-certainty evidence that nicotine-containing e-cigarettes increase smoking quit rates compared with nicotine replacement therapy (NRT), and moderate-certainty evidence that they probably increase quit rates compared with non-nicotine e-cigarettes. The primary limitation of the current evidence base is imprecision in some comparisons and uncertainty regarding safety outcomes, largely due to the relatively small number of randomized controlled trials (RCTs), many of which report low event rates. Additional large-scale RCTs with longer follow-up are underway and remain necessary [67]. Although e-cigarettes are generally considered less harmful than combustible cigarettes, many adults in the United States who use e-cigarettes continue to smoke cigarettes concurrently (dual use). Reduction in e-cigarette use is common and can be achieved by switching to lower-nicotine products or by decreasing frequency of use. Among dual users who were initially unmotivated to quit smoking, interventions focused on reducing e-cigarette use increased treatment engagement, while switching to low-nicotine e-cigarettes was associated with increased cigarette quit attempts at four-week follow-up [68].

These cessation efficacy data should, however, be interpreted in parallel with the cardiovascular safety signals reviewed above. While nicotine-containing e-cigarettes may increase smoking abstinence compared with NRT in the general population, the available evidence on long-term cardiovascular outcomes remains limited and is largely observational, with potential confounding by prior smoking intensity, dual use, and device heterogeneity. Consequently, efficacy for smoking cessation does not necessarily imply cardiovascular safety, particularly in patients with established cardiovascular disease or high baseline risk. For cardiac patients, the clinical challenge is therefore a risk–benefit trade-off: switching completely from combustible cigarettes to exclusive e-cigarette use may reduce exposure to some combustion-related toxicants, yet e-cigarette aerosols can still exert adverse vascular, autonomic, thrombotic, and pro-arrhythmic effects, and prospective data on hard cardiovascular endpoints are insufficient to provide reassurance.

Certain professional societies adopt a harm reduction framework, considering these products as alternatives owing to their reportedly lower toxic content compared to combustible tobacco. However, scientific evidence for these recommendations remains insufficient, particularly regarding long-term effects, and should be critically examined alongside evolving tobacco industry marketing [69].

In clinical practice, cardiologists should prioritize evidence-based smoking cessation strategies with established safety and efficacy profiles (behavioral support plus approved pharmacotherapies such as varenicline, bupropion, or NRT). If a patient with cardiovascular disease refuses these options or has repeatedly failed conventional cessation therapies, a harm-reduction approach may be discussed cautiously and individualized, emphasizing (i) the goal of complete cessation of combustible tobacco as the primary priority, (ii) avoidance of dual use, which appears to confer little or no cardiovascular benefit, and (iii) structured follow-up with a clear plan to taper and discontinue all nicotine products when feasible. Shared decision-making should explicitly communicate that current evidence does not confirm long-term cardiovascular safety of e-cigarettes, and that any potential benefit is most plausible only when e-cigarettes replace—not supplement—combustible smoking.

## 7. Conclusions

In our opinion, e-cigarettes should not be recommended as a tool for smoking cessation, as many individuals continue vaping long after quitting combustible tobacco. In 2015, e-cigarettes were already considered a public health threat and not a validated aid for quitting smoking or reducing tobacco risk, given the lack of solid evidence and regulatory oversight regarding their quality, effectiveness, and safety [7]. A decade later, in 2025, the body of evidence has grown, but the message remains one of caution. Table 1 summarizes representative studies investigating associations between electronic cigarette (e-cigarette) use (exposure) and cardiovascular outcomes, including coronary artery disease, myocardial infarction, blood pressure changes, endothelial dysfunction, arrhythmias, and heart failure. Comparators include non-users, former smokers, or conventional cigarette smokers, depending on study design. Effect estimates (e.g., odds ratios [OR], hazard ratios [HR], or physiological changes) are reported as described in the original studies (Exposure groups: current e-cigarette users or experimental exposure to e-cigarette aerosol. Comparators: non-users, former smokers, or conventional cigarette smokers. Outcomes: clinical cardiovascular events, physiological changes, or vascular function measures. Effect estimates: OR = odds ratio; HR = hazard ratio; CI = confidence interval).

Cardiologists should exercise prudence and advise patients against e-cigarette use. The decision of a patient with cardiovascular disease to quit smoking by switching to e-cigarettes does not obligate clinicians to endorse these devices. Some researchers [69] recommend e-cigarette to be excluded from standard cessation recommendations in clinical guidelines. A multi-disciplinary approach, free from industry influence and focused on safeguarding young people, remains the most effective way to preserve the hard-earned progress achieved in tobacco control. Instead, patients should be offered alternatives with demonstrated efficacy, safety, and quality, such as approved pharmacological therapies and cognitive–behavioral interventions. The European Respiratory Society (ERS) maintains that all nicotine and tobacco products are highly addictive and harmful, and complete cessation of all nicotine products remains the goal to reduce tobacco-related morbidity and mortality [70]. The ERS Tobacco Control Committee further emphasizes the lack of independent evidence to support the tobacco industry’s “harm reduction” claims.

The European Union continues to prioritize tobacco control and has set the goal of achieving a “tobacco-free generation” by 2040. Accordingly, the ERS does not recommend any lung-damaging products and rejects harm reduction as a population-level strategy. Complementary recommendations from the European Association of Preventive Cardiology (EAPC) include: (a) mounting evidence shows e-cigarettes are harmful, including to the cardiovascular system; (b) smokers may use e-cigarettes as a supplement rather than a substitute for tobacco; (c) robust evidence of efficacy for smoking cessation is lacking; and (d) e-cigarettes appear to displace evidence-based cessation methods and clinics [71].

In the United States, the Food and Drug Administration (FDA) has yet to implement strict regulations on the distribution of e-cigarettes, particularly the newest (fourth-generation) devices, and must develop strategies to address the associated health risks. Another concern is the lack of transparency in research: one-third of published studies on e-cigarettes fail to include conflict-of-interest disclosures, a proportion even higher in editorials, news articles, and other non-original reports [72]. Journal editors and reviewers should scrutinize funding sources and potential biases when evaluating such publications.

Ultimately, after extensive clinical and experimental research, clinicians should consistently inquire about and document e-cigarette use in their patients. This practice is essential for accurately assessing health risks and guiding appropriate preventive and therapeutic strategies [73].

Given the growing body of experimental and epidemiological evidence, clinicians should approach e-cigarette use with caution and prioritize evidence-based smoking cessation strategies while awaiting more definitive long-term cardiovascular safety data.

## 8. Limitations

This review has several limitations. First, it is a narrative review, and not a systematic one; therefore, it may not include every study published on the topic. Second, the available human evidence on the cardiovascular effects of e-cigarettes is largely observational and may be confounded by dual use, prior smoking history, and device heterogeneity. Third, as noted above, potential conflicts of interest in some studies could influence reported outcomes. Finally, the rapidly evolving technology of vaping devices and e-liquids limits the generalizability of earlier findings to newer products. Despite these limitations, the reviewed literature consistently indicates that e-cigarette use is not free from cardiovascular risk.

It is important to note that some published studies in this field have potential conflicts of interest, including financial or employment relationships with the tobacco or e-cigarette industry. Such affiliations may influence study design, interpretation, or reporting of results. To mitigate this bias, we prioritized peer-reviewed studies from independent or academic research groups whenever possible and interpreted findings with caution, particularly when funding disclosures were incomplete or absent.

## Figures and Tables

**Table 1:** Evidence from published human and experimental studies evaluating the cardiovascular effects of electronic cigarette exposure.

Outcome	Key Findings	Study Type	Reported Effect	References
Coronary artery disease	Reduced FMD; ↑ oxidative stress	Observational	Endothelial dysfunction similar to smoking	[14,17,24]
Myocardial infarction	Increased odds of myocardial infarction among current e-cigarette users compared with non-users, with a higher risk observed among dual users (e-cigarette + combustible cigarettes)	Meta-analysis of observational studies	OR 1.44 (95% CI 1.22–1.74) for MI in e-cigarette users vs non-users; dual users show substantially higher risk	[31,32,35]
Arrhythmias	Sympathoexcitatory, QT prolongation	Cross-sectional	↑ HR/BP; pro-arrhythmic	[42,44,46]
Hypertension	Nicotine-induced sympathetic activation	Observational	↑ BP, ↑ HR	[54]
Heart failure	19% increased risk	Cohort	HF pEF association	[25,49]

Abbreviations: MI—myocardial infarction; OR—odds ratio; CI—confidence interval; HR—heart rate; BP—blood pressure; HF—heart failure; HFpEF—heart failure with preserved ejection fraction; FMD—flow-mediated dilation.

## Data Availability

No new data were created.

## References

[1] Toledo E.F.V., Simões I.F., Farias M.T.D., Minho L.A.C., Conceição J.D.L., Santos W.N.L.D., Mesquita P.R.R.D., Júnior A.D.F.S. (2025). A Comprehensive Review of the Harmful Compounds in Electronic Cigarettes. Toxics.

[2] Lee W.H., Ong S.G., Zhou Y., Tian L., Bae H.R., Baker N., Whitlatch A., Mohammadi L., Guo H., Nadeau K.C. (2019). Modeling Cardiovascular Risks of E-Cigarettes With Human-Induced Pluripotent Stem Cell-Derived Endothelial Cells. J. Am. Coll. Cardiol..

[3] U.S. Department of Health and Human Services (2016). E-Cigarette Use Among Youth and Young Adults: A Report of the Surgeon General.

[4] Gaddey H.L., Dakkak M., Jackson N.M. (2022). Smoking Cessation Interventions. Am. Fam. Physician.

[5] World Health Organization Regional Office for Europe (2024). Prevalence of Tobacco and E-Cigarette Use by Young People: Factsheet 2024.

[6] Adebisi Y.A., Jimoh N.D., Ngoma C. (2025). ‘E-cigarette smoking’ is a misleading term: A critical review of its use in academic literature. Intern. Emerg. Med..

[7] Bobadilla J.F., Dalmau R., Saltó E. (2015). Cardiologists and electronic cigarettes. Rev. Esp. Cardiol..

[8] Goniewicz M.L., Knysak J., Gawron M., Kosmider L., Sobczak A., Kurek J., Prokopowicz A., Jablonska-Czapla M., Rosik-Dulewska C., Havel C. (2014). Levels of selected carcinogens and toxicants in vapour from electronic cigarettes. Tob. Control.

[9] Kaur G., Muthumalage T., Rahman I. (2018). Mechanisms of toxicity and biomarkers of flavoring and flavor enhancing chemicals in emerging tobacco and non-tobacco products. Toxicol. Lett..

[10] Hansson J., Galanti M.R., Hergens M.P., Fredlund P., Ahlbom A., Alfredsson L., Bellocco R., Eriksson M., Hallqvist J., Hedblad B. (2012). Use of snus and acute myocardial infarction: Pooled analysis of eight prospective observational studies. Eur. J. Epidemiol..

[11] Skotsimara G., Antonopoulos A.S., Oikonomou E., Siasos G., Ioakeimidis N., Tsalamandris S., Charalambous G., Galiatsatos N., Vlachopoulos C., Tousoulis D. (2019). Cardiovascular effects of electronic cigarettes: A systematic review and meta-analysis. Eur. J. Prev. Cardiol..

[12] Abouassali O., Chang M., Chidipi B., Martinez J.L., Reiser M., Kanithi M., Soni R., McDonald T.V., Herweg B., Saiz J. (2021). In vitro and in vivo cardiac toxicity of flavored electronic nicotine delivery systems. Am. J. Physiol. Heart Circ. Physiol..

[13] Ramalingam A.R., Kucera C., Srivastava S., Paily R., Stephens D., Lorkiewicz P., Wilkey D.W., Merchant M., Bhatnagar A., Carll A.P. (2025). Acute and persistent cardiovascular effects of menthol e-cigarettes in mice. J. Am. Heart Assoc..

[14] Carnevale R., Sciarretta S., Violi F., Nocella C., Loffredo L., Perri L., Peruzzi M., Marullo A.G., De Falco E., Chimenti I. (2016). Acute impact of tobacco versus electronic cigarette smoking on oxidative stress and vascular function. Chest.

[15] Moheimani R.S., Bhetraratana M., Yin F., Peters K.M., Gornbein J., Araujo J.A., Middlekauff H.R. (2017). Increased cardiac sympathetic activity and oxidative stress in habitual electronic cigarette users: Implications for cardiovascular risk. JAMA Cardiol..

[16] Moheimani R.S., Bhetraratana M., Peters K.M., Yang B.K., Yin F., Gornbein J., Araujo J.A., Middlekauff H.R. (2017). Sympathomimetic effects of acute e-cigarette use: Role of nicotine and non-nicotine constituents. J. Am. Heart Assoc..

[17] Kennedy C.D., van Schalkwyk M.C.I., McKee M., Pisinger C. (2019). The cardiovascular effects of electronic cigarettes: A systematic review of experimental studies. Prev. Med..

[18] Espinoza-Derout J., Shao X.M., Lao C.J., Hasan K.M., Rivera J.C., Jordan M.C., Echeverria V., Roos K.P., Sinha-Hikim A.P., Friedman T.C. (2022). Electronic cigarette use and the risk of cardiovascular diseases. Front. Cardiovasc. Med..

[19] Tsai M., Byun M.K., Shin J., Crotty Alexander L.E. (2020). Effects of e-cigarettes and vaping devices on cardiac and pulmonary physiology. J. Physiol..

[20] Li X., Yuan L., Wang F. (2024). Health outcomes of electronic cigarettes. Chin. Med. J..

[21] Jiang C., Xie N., Sun T., Ma W., Zhang B., Li W. (2020). Xanthohumol Inhibits TGF-β1-Induced Cardiac Fibroblasts Activation via Mediating PTEN/Akt/mTOR Signaling Pathway. Drug Des. Devel. Ther..

[22] Farsalinos K.E., Polosa R., Cibella F., Niaura R. (2019). Is e-cigarette use associated with coronary heart disease and myocardial infarction? Insights from the 2016 and 2017 National Health Interview Surveys. Ther. Adv. Chronic Dis..

[23] Osei A.D., Mirbolouk M., Orimoloye O.A., Dzaye O., Uddin S.M.I., Benjamin E.J., Hall M.E., DeFilippis A.P., Stokes A., Bhatnagar A. (2019). Association between e-cigarette use and cardiovascular disease among never and current combustible-cigarette smokers. Am. J. Med..

[24] Berlowitz J.B., Xie W., Harlow A.F., Hamburg N.M., Blaha M.J., Bhatnagar A., Benjamin E.J., Stokes A.C. (2022). E-cigarette use and risk of cardiovascular disease: A longitudinal analysis of the PATH Study (2013–2019). Circulation.

[25] American College of Cardiology (2024). Study Links E-Cigarette Use with Higher Risk of Heart Failure. https://www.acc.org/About-ACC/Press-Releases/2024/04/01/21/51/study-links-e-cigarette-use-with-higher-risk-of-heart-failure.

[26] Chen C., Huo C., Mattey-Mora P.P., Bidulescu A., Parker M.A. (2024). Assessing the association between e-cigarette use and cardiovascular disease: A meta-analysis of exclusive and dual use with combustible cigarettes. Addict. Behav..

[27] American Heart Association (2022). Smoking Both Traditional and E-Cigarettes may Carry Same Heart Risks as Cigarettes Alone. https://www.heart.org/en/news/2022/05/06/smoking-both-traditional-and-e-cigarettes-may-carry-same-heart-risks-as-cigarettes-alone.

[28] Wang J.B., Olgin J.E., Nah G., Vittinghoff E., Cataldo J.K., Pletcher M.J., Marcus G.M. (2018). Cigarette and e-cigarette dual use and risk of cardiopulmonary symptoms in the Health eHeart Study. PLoS ONE.

[29] Benowitz N.L., Burbank A.D. (2016). Cardiovascular toxicity of nicotine: Implications for electronic cigarette use. Trends Cardiovasc. Med..

[30] Busch A.M., Leavens E.L., Wagener T.L., Buckley M.L., Tooley E.M. (2016). Prevalence, reasons for use, and risk perception of electronic cigarettes among post-acute coronary syndrome smokers. J. Cardiopulm. Rehabil. Prev..

[31] Ashraf M.T., Shaikh A., Khan M.K.S., Uddin N., bin Kashif M.A., Rizvi S.H.A., Khalid H., Sam S.J., Sohail A. (2023). Association between e-cigarette use and myocardial infarction: A systematic review and meta-analysis. Egypt. Heart J..

[32] Sharma A., Gupta I., Venkatesh U., Singh A.K., Golamari R., Arya P. (2023). E-cigarettes and myocardial infarction: A systematic review and meta-analysis. Int. J. Cardiol..

[33] Qasim H., Karim Z.A., Silva-Espinoza J.C., Khasawneh F.T., Rivera J.O., Ellis C.C., Bauer S.L., Almeida I.C., Alshbool F.Z. (2018). Short-term e-cigarette exposure increases the risk of thrombogenesis and enhances platelet function in mice. J. Am. Heart Assoc..

[34] Nocella C., Biondi-Zoccai G., Sciarretta S., Peruzzi M., Pagano F., Loffredo L., Pignatelli P., Bullen C., Frati G., Carnevale R. (2018). Impact of tobacco versus electronic cigarette smoking on platelet function. Am. J. Cardiol..

[35] Alzahrani T., Pena I., Temesgen N., Glantz S.A. (2018). Association between electronic cigarette use and myocardial infarction. Am. J. Prev. Med..

[36] Alzahrani T., Glantz S.A. (2019). Adding data from 2015 strengthens the association between e-cigarette use and myocardial infarction. Am. J. Prev. Med..

[37] Nazir A., Shetty Ujjar S., Seddiki M.O., Jheinga M., Fan L. (2025). Smoking cessation strategies after acute coronary syndrome. J. Clin. Med..

[38] Lindson N., Theodoulou A., Ordóñez-Mena J.M., Fanshawe T.R., Sutton A.J., Livingstone-Banks J., Hajizadeh A., Zhu S., Aveyard P., Freeman S.C. (2023). Pharmacological and electronic cigarette interventions for smoking cessation in adults: Component network meta-analyses. Cochrane Database Syst. Rev..

[39] Savko C., Esquer C.A.S., Molinaro C., Rokaw S., Shain A.G., Jaafar F., Wright M.K., Phillips J.A., Hopkins T., Mikhail S. (2025). Myocardial infarction injury is exacerbated by nicotine in vape aerosol exposure. J. Am. Heart Assoc..

[40] Garcia P.D., Gornbein J.A., Middlekauff H.R. (2020). Cardiovascular autonomic effects of electronic cigarette use: A systematic review. Clin. Auton. Res..

[41] Antoniewicz L., Brynedal A., Hedman L., Lundbäck M., Bosson J.A. (2019). Acute effects of electronic cigarette inhalation on the vasculature and the conducting airways. Cardiovasc. Toxicol..

[42] Kundu A., Feore A., Sanchez S., Abu-Zarour N., Sutton M., Sachdeva K., Seth S., Schwartz R., Chaiton M. (2025). Cardiovascular health effects of vaping e-cigarettes: A systematic review and meta-analysis. Heart.

[43] Carll A.P., Arab C., Salatini R., Miles M.D., Nystoriak M.A., Fulghum K.L., Riggs D.W., Shirk G.A., Theis W.S., Talebi N. (2022). E-cigarettes and their lone constituents induce cardiac arrhythmia and conduction defects in mice. Nat. Commun..

[44] Jones C.A., Wallace M.J., Bandaru P., Woodbury E.D., Mohler P.J., Wold L.E. (2023). E-cigarettes and arrhythmogenesis: A comprehensive review of pre-clinical studies and their clinical implications. Cardiovasc. Res..

[45] Conklin D.J., Ogunwale M.A., Chen Y., Theis W.S., Nantz M.H., Fu X.A., Chen L.-C., Riggs D.W., Lorkiewicz P., Bhatnagar A. (2018). Electronic cigarette-generated aldehydes: The contribution of e-liquid components to their formation and the use of urinary aldehyde metabolites as biomarkers of exposure. Aerosol Sci. Technol..

[46] Cutler M.J., Jeyaraj D., Rosenbaum D.S. (2011). Cardiac electrical remodeling in health and disease. Trends Pharmacol. Sci..

[47] Prochaska J.J., Vogel E.A., Benowitz N. (2022). Nicotine delivery and cigarette equivalents from vaping a JUULpod. Tob. Control.

[48] Bolaji O., Bahar A.R., Bahar Y., Areoye G., Osei A.D., Adeboye A.A., Mazimba S. (2025). Vaping and heart failure: A narrative review. Cardiol. Rev..

[49] Bene-Alhasan Y., Mensah S., Almaadawy O., Dwumah-Agyen M., Pingili A., Mlilo M., Osei A.D. (2024). Electronic nicotine product use is associated with incident heart failure—The All of Us Research Program. J. Am. Coll. Cardiol..

[50] Zhu Y., Zhang Q., Wang Y., Liu W., Zeng S., Yuan Q., Zhang K. (2025). Identification of Necroptosis and Immune Infiltration in Heart Failure Through Bioinformatics Analysis. J. Inflamm. Res..

[51] Hasan K.M., Friedman T.C., Parveen M., Espinoza-Derout J., Bautista F., Razipour M.M., Shao X.M., Jordan M.C., Roos K.P., Mahata S.K. (2020). Electronic cigarettes cause alteration in cardiac structure and function in diet-induced obese mice. PLoS ONE.

[52] Espinoza-Derout J., Hasan K.M., Shao X.M., Jordan M.C., Sims C., Lee D.L., Sinha S., Simmons Z., Mtume N., Liu Y. (2019). Chronic intermittent electronic cigarette exposure induces cardiac dysfunction and atherosclerosis in apolipoprotein-E knockout mice. Am. J. Physiol. Heart Circ. Physiol..

[53] Gordan R., Gwathmey J.K., Xie L.H. (2015). Autonomic and endocrine control of cardiovascular function. World J. Cardiol..

[54] Lee J., Rodriguez C.J., Daviglus M.L., Kaplan R., Isasi C.R., Sotres-Alvarez D., Blaha M.J., Mok Y., Perreira K.M., Oren E. (2026). Electronic Cigarette Use and Risk of Hypertension: The Hispanic Community Health Study/Study of Latinos (HCHS/SOL). JACC Adv..

[55] Vindhyal M.R., Ndunda P., Munguti C., Vindhyal S., Okut H. (2019). Impact on cardiovascular outcomes among e-cigarette users: A review from National Health Interview Surveys. J. Am. Coll. Cardiol..

[56] Tansawet A., Anothaisintawee T., Boonmanunt S.W., Pornsuriyasak P., Sukhato K., Chawala N., Inpithuk P., Savigamin C., Liampeng S., Attia J. (2025). Electronic cigarettes and cardiovascular diseases: An updated sys-tematic review and network meta-analysis. Tob. Induc. Dis..

[57] Biondi-Zoccai G., Sciarretta S., Bullen C., Nocella C., Violi F., Loffredo L., Pignatelli P., Perri L., Peruzzi M., Marullo A.G.M. (2019). Acute effects of heat-not-burn, electronic vaping, and traditional tobacco combustion cigarettes: The SUR-VAPES 2 randomized trial. J. Am. Heart Assoc..

[58] Balakumar P., Kaur J. (2009). Arsenic exposure and cardiovascular disorders: An overview. Cardiovasc. Toxicol..

[59] Wang Z., Liu J., Chen Y., Tang Y., Chen T., Zhou C., Wang S., Chang R., Chen Z., Yang W. (2025). From physiology to pathology: Emerging roles of GPER in cardiovascular disease. Pharmacol. Ther..

[60] Guo B., Jiang X., Zhu L., He X. (2026). Exploring the Diagnostic Potential of Core Targets of 6PPD and Its Metabolite 6PPD-Q in Cardiovascular Diseases: An Integrated Analysis Based on Network Toxicology, Molecular Docking, and In Vitro Validation. J. Appl. Toxicol..

[61] Institute for Global Tobacco Control (2019). Country Laws Regulating E-Cigarettes.

[62] Cheng K.W., Chaloupka F.J., Shang C., Ngo A., Fong G.T., Borland R., Heckman B.W., Levy D.T., Cummings K.M. (2019). Prices, use restrictions and electronic cigarette use—Evidence from Wave 1 (2016) US data of the ITC Four Country Smoking and Vaping Survey. Addiction.

[63] Article 20 of the Tobacco Products Directive (2014/40/EU) Lays Down Rules for Electronic Cigarettes Sold as Consumer Products in the EU. https://health.ec.europa.eu/tobacco/product-regulation/electronic-cigarettes_en?utm_source=chatgpt.com.

[64] Koprivnikar H., Carnicer-Pont D., López A.M., González-Marrón A., Sæbø G., Gallus S., Possenti I., Lambrou A., Papachristou E., Pénzes M. (2025). Recommendations for updating regulations on advertising, promotion and sponsorship of tobacco and nicotine products in the European Union. Tob. Prev. Cessat..

[65] Jang Y., Shaw J., Wackowski O.A., Noar S.M. (2025). Effectiveness of text-only e-cigarette warnings: A meta-analysis. JAMA Intern. Med..

[66] Butler A.R., Lindson N., Livingstone-Banks J., Notley C., Turner T., Rigotti N.A., Fanshawe T.R., Dawkins L., Begh R., Wu A.D. (2025). Interventions for quitting vaping. Cochrane Database Syst. Rev..

[67] Lindson N., Butler A.R., McRobbie H., Bullen C., Hajek P., Begh R., Theodoulou A., Notley C., Rigotti N.A., Turner T. (2024). Electronic cigarettes for smoking cessation. Cochrane Database Syst. Rev..

[68] Feinstein M.J., Skelly J., Higgins S., Klemperer E. (2024). W34-Treatment Engagement, Cigarette Smoking, and Quit Attempts one Month After a Randomized Controlled Trial of E-Cigarette Reduction Among Dual Users. Drug Alcohol. Depend..

[69] Yıldız E., Kılınç O., Çuhadaroğlu Ç. (2026). Echoes of the past: Are e-cigarettes the new “light” cigarettes?. Balk. Med. J..

[70] Chen D.T., Grigg J., Filippidis F.T., Tobacco Control Committee of the European Respiratory Society (2024). European Respiratory Society statement on novel nicotine and tobacco products, their role in tobacco control and “harm reduction.”. Eur. Respir. J..

[71] Kavousi M., Pisinger C., Barthelemy J.-C., De Smedt D., Koskinas K., Marques-Vidal P., Panagiotakos D., Prescott E.B., Tiberi M., Vassiliou V.S. (2021). Electronic cigarettes and health with special focus on cardiovascular effects: Position paper of the European Association of Preventive Cardiology (EAPC). Eur. J. Prev. Cardiol..

[72] Martínez C., Fu M., Galán I., Pérez-Rios M., Martínez-Sánchez J.M., López M.J., Sureda X., Montes A., Fernández E. (2018). Conflicts of interest in research on electronic cigarettes. Tob. Induc. Dis..

[73] Neczypor E.W., Mears M.J., Ghosh A., Sassano M.F., Gumina R.J., Wold L.E., Tarran R. (2022). E-cigarettes and cardiopulmonary health: Review for clinicians. Circulation.

